# KIR-HLA Functional Repertoire Influences Trastuzumab Efficiency in Patients With HER2-Positive Breast Cancer

**DOI:** 10.3389/fimmu.2021.791958

**Published:** 2022-01-12

**Authors:** Elena Muraro, Mariangela De Zorzi, Gianmaria Miolo, Davide Lombardi, Simona Scalone, Simon Spazzapan, Samuele Massarut, Tiziana Perin, Riccardo Dolcetti, Agostino Steffan, Valli De Re

**Affiliations:** ^1^ Immunopathology and Cancer Biomarkers Units, Department of Translational Research, Centro di Riferimento Oncologico di Aviano (CRO), Istituto di Ricovero e Cura a Carattere Scientifico (IRCCS), Aviano, Italy; ^2^ Medical Oncology and Cancer Prevention Unit, Centro di Riferimento Oncologico di Aviano (CRO), IRCCS, Aviano, Italy; ^3^ Breast Surgery Unit, Centro di Riferimento Oncologico di Aviano (CRO Aviano), IRCCS, National Cancer Institute, Aviano, Italy; ^4^ Pathology Unit, Centro di Riferimento Oncologico di Aviano (CRO Aviano), IRCCS, National Cancer Institute, Aviano, Italy; ^5^ Centre for Cancer Immunotherapy, Peter MacCallum Cancer Centre, Melbourne, VIC, Australia; ^6^ Sir Peter MacCallum Department of Oncology, The University of Melbourne, VIC, Australia; ^7^ Department of Microbiology and Immunology, The University of Melbourne, VIC, Australia; ^8^ Faculty of Medicine, The University of Queensland Diamantina Institute, Brisbane, QLD, Australia

**Keywords:** KIR, HLA, breast cancer, trastuzumab, ADCC

## Abstract

Trastuzumab induced a high rate of pathological Complete Response (pCR) in patients affected by locally advanced HER2-positive Breast Cancer (HER2-BC), by exploiting immune-mediated mechanisms as Antibody-Dependent Cell Cytotoxicity (ADCC) involving Natural Killer (NK) cells. Host’s immune genetics could influence the response to therapy, through the expression of variants that characterize NK receptors involved in ADCC effectiveness. Killer cell immunoglobin-like receptors (KIRs) modulate NK cell activity through their binding to class-I Human Leukocyte Antigens (HLA). The impact of the KIR/HLA repertoire in HER2-BC is under study. We characterized KIR genotypes of 36 patients with locally advanced HER2-BC treated with neoadjuvant chemotherapy including trastuzumab. We monitored pCR achievement before surgery and Disease-Free Survival (DFS) and Overall Survival (OS) after adjuvant therapy. HLA, and Fc gamma receptor IIIa (FcγR3A) and IIa (FcγR2A) were genotyped through targeted PCR and Sanger sequencing in 35/36 patients. The KIR-HLA combinations were then described as functional haplotypes and divided in two main categories as inhibitory tel A and stimulatory tel B. Trastuzumab-dependent ADCC activity was monitored with an *in vitro* assay using a HER2-BC model and patients’ NK cells.We observed a higher frequency of KIR activators in patients who achieved a pCR compared to partial responders. During the study of functional haplotypes, individuals carrying a tel B haplotype showed greater ADCC efficiency than tel A cases. In subjects with the tel A haplotype the presence of the favorite V allele in FcγR3A receptor improved their low ADCC levels. Regardless of the haplotypes detected, the presence of KIR3DL2/HLA-A03 or A11 was always associated with the FcγR3A V allele, and therefore correlated with greater ADCC efficiency. However, this particular KIR receptor appeared to harm DFS and OS. Indeed, patients with tel B haplotype without KIR3DL2/HLA-A03 or A11 showed a better outcome. Our data, although preliminary, suggested a potential predictive role for KIR haplotype tel B, in identifying patients who achieve a pCR after neoadjuvant treatment with trastuzumab, and supported a negative prognostic impact of KIR3DL2/HLA-A03 or A11 in the adjuvant setting.

## Introduction

At present, Neoadjuvant Chemotherapy for the treatment of locally advanced HER2-positive Breast Cancer (BC) includes trastuzumab (Herceptin), a humanized IgG1 monoclonal antibody (mAb) targeting human epidermal growth factor receptor-2 (HER2). The receptor triggers downstream signal by forming heterodimers with other HER family members [HER1 (also named Epidermal Growth Factor, EGFR), HER3 and HER4], leading to auto- and/or trans-phosphorylation of definite tyrosine residues within the cytoplasmatic domain of these receptors, thus providing signaling cascades promoting cell proliferation and survival. By blocking the extracellular ligand domain of HER2, trastuzumab antagonizes the functional activity of the receptor and the cleavage of the extracellular domain of HER2 leading to a downmodulation of the function and formation of HER heterodimers. In addition, accumulating evidence suggested a relevant role of trastuzumab in the engagement of immune cells, such as Natural Killer (NK) cells expressing the Fc receptor CD16, which through antibody-mediated cellular cytotoxicity (ADCC) may result in the killing of target cells expressing HER2 (1). Trastuzumab is used in about 20 to 30% of BC, achieving high rates of pathological Complete Response (pCR) (2). The achievement of a pCR after NC has been associated with long-term survival, thus becoming a surrogate endpoint and a potential prognostic biomarker (3). Moreover, trastuzumab treatment contributed to the induction of an improved Disease-Free Survival (DFS) when used in the adjuvant setting for 1 year (4). We and others showed that a higher proficiency of both, innate and adaptive host immunity, characterized patients with BC achieving a pCR compared to partial responders (5–7). This evidence suggests that a better knowledge of the host immunity in this setting may be relevant for the identification of suitable biomarkers able to predict pCR and with possible prognostic significance.

Several studies have reported a correlation between clinical response and polymorphisms of Fc gamma receptor IIIa (FcγR3A, or CD16A) on NK cells, which recognizes the Fc portion of mAbs mediating ADCC as trastuzumab, bound to the tumor cells (8). As we noticed previously, patients carrying the FcγR3A-V-allele (9), responsible for a stronger affinity to Fc, showed an increased ADCC activity compared to the F allele (10, 11). The importance of ADCC effect in BC treated with trastuzumab was also demonstrated through the evidence of a dramatic reduction of its efficacy in knockout mice for FcγR3A (12).

NK cell activation depends also on the interaction with other receptors like the killer cell immunoglobin-like receptors (KIRs), which bind the peptide-binding region of several class-I Human Leukocyte Antigens (HLA class-I) (13). Several studies have demonstrated that the absence of HLA class-I on tumor cells, or their decreased expression, damped the HLA-inhibitory KIRs interaction thus improving NK cell activation, including the ADCC activity (14, 15). KIR positivity is acquired late during NK cell maturation, resulting expressed mainly by the most mature CD16bright CD56dim NK cells. KIR family members consist of 13 activating and inhibitory genes and 2 pseudogenes (KIR2DP1, and KIR3DP1), which separate the KIR genotype into two halves: the centromeric and the telomeric half. KIR gene region shows a difference in KIR gene contents and polymorphisms, and functions only in the presence of specific cognate HLA ligands, currently still not completely described (16), thus leading to a vast KIR functional repertoire where the inhibitory variants are prevalent (16–18). The number and type of KIR genes define different KIR haplotypes, which have been classified into two main groups, termed “A” and “B” genotypes (16). The A genotype is mainly constituted of the inhibitory receptors and the only activating 2DS4 KIR, while the B genotype shows a variable number of activating KIR.

Accumulating evidence suggested that different KIR/HLA gene combinations and HLA expression levels can influence tumor prognosis and treatment response (19). Studies employing the anti-KIR mAb lirilumab in combination with rituximab demonstrated a higher ADCC efficiency mediated by NK cells against lymphoma *in vitro* and *in vivo*, due to the interruption of the binding between inhibitory KIRs and their ligand and the consequent inhibitory signal (20, 21). Interestingly, using KIR-ligand-mismatched NK subsets Ehlers and colleagues demonstrated a stronger degranulation of NK cells against BC cells in the presence of trastuzumab (1). Moreover, Terszowski et al. demonstrated that both KIR/HLA interaction and the FcγR3A V-allele may act synergistically to improve NK cell activation *in vitro* (11, 22). However, the influence of particular KIR haplotypes in the efficiency of neoadjuvant trastuzumab for the treatment of BC remains unaddressed (10).

On these grounds, the present study aimed at exploring the possible correlation of KIR/HLA haplotypes with the induction of a pCR after NC in a cohort of patients affected by locally advanced BC overexpressing HER2 and treated with trastuzumab. We focused in particular on the efficiency of the ADCC mediated by NK cells and assessed the potential synergic impact of KIR haplotype and FcγR3A polymorphisms. Moreover, we evaluated whether the identification of such a predictive haplotype could have a prognostic impact in the same cohort of patients also in the follow-up after surgery and adjuvant chemotherapy.

## Materials and Methods

### Patients and Biological Samples

The present study analyzed the impact of genetic KIR/HLA combination performed in blood samples obtained from 36 patients affected by locally advanced HER2-overexpressing breast carcinoma (defined as not susceptible of conservative surgery at diagnosis; UICC, International Union Against Cancer, stage II to III) and included in the phase II CRO Clinical Trial, NCT02307227 (9). HER2 status was assessed by immunohistochemistry (IHC) and chromogenic *in situ* hybridization or fluorescence *in situ* hybridization in the case of IHC 2+. Almost all patients (34/36, 94.4%) showed overexpressed HER2 oncoprotein with a strong IHC score(IHC 3+); 2 patients (2/36, 5.6%) showed a weakly positive IHC score (IHC 2+) but were ISH positive for *HER2* gene amplification. All patients had the following clinical features: Eastern Cooperative Oncology Group (ECOG) performance status of 0 or 1; baseline left ventricular ejection fraction greater than 50%; adequate organ function (bone marrow function: neutrophils ≥2.0 × 10^9^/L, platelets ≥120 × 10^9^/L; liver function: serum bilirubin <1.5 times the upper limit of normal (ULN), transaminases <2.5 times ULN, alkaline phosphatase ≤2.5 times ULN, serum creatinine <1.5 times ULN). Patients received neoadjuvant chemotherapy with trastuzumab (loading dose 4 mg/kg intravenously, then 2 mg/kg weekly) and concomitant weekly Paclitaxel (80 mg/m2) for 3 cycles, followed by clinical evaluation and, in case of clinical response, 3 more cycles to obtain a pCR. After neoadjuvant chemotherapy, patients underwent primary surgery (mastectomy or conservative treatment) as well as axillary node dissection. In selected patients based on tumor pathological features, post-mastectomy radiation was performed to the chest wall and in addition at the axillary region when they showed more than 3 positive lymph nodes. After surgery, adjuvant chemotherapy with 3 more cycles of trastuzumab and paclitaxel (12 weeks) was planned, and trastuzumab alone every 3 weeks was continued for a total of 1 year, together with hormonal therapy for 5 years in the case of Estrogen Receptor (ER) and/or Progesterone Receptor (PgR) positive tumor. The instrumental evaluation was performed at baseline and every 12 weeks. Patients’ follow-up was monitored for a maximum of 120 months. This study was conducted according to the ethical principles of the Declaration of Helsinki and approved by the local Ethical Committee (Comitato Etico Indipendente del CRO di Aviano, May 29, 2006). Written informed consent was obtained from all patients.

Blood samples were collected from each patient at diagnosis, at the 12^th^ and 24^th^ week of neoadjuvant treatment, and 2, 6, and 12 months of follow-up. Samples were transported at room temperature and processed within 5 hours. Genomic DNA was purified using the DNA extraction kit (EZ1 DNA Blood 350 μl kit, Qiagen, Valencia, CA) from blood samples obtained at diagnosis from all patients. Peripheral blood mononuclear cells (PBMCs) were freshly isolated from heparinized blood of patients by Ficoll-Hypaque gradient (Lymphoprep, Fresenius Kabi Norge Halden) using standard gradient separation. Cells were washed in PBS (Biomerieux), counted using Trypan blue (viability >90%), and viably frozen (90% heat-inactivated Fetal Bovine Serum [Gibco^®^, Life Technologies] and 10% DMSO) at −80°C for 24 h and then in liquid nitrogen until use. After thawing in RPMI-1640 medium (Sigma-Aldrich) with 3 μg/ml Deoxyribonuclease (Sigma-Aldrich), cells were washed in PBS (Biomerieux) and counted again to check the viability.

### ADCC Assay, Flow Cytometry and Analysis of Fcγ Receptor Polymorphisms

The trastuzumab-dependent ADCC efficiency, the NK cells number quantification, and the immunogenetic analysis of FcγR polymorphisms were evaluated as already described (9). Briefly, the cytotoxic activity mediated by trastuzumab was quantified in a Calcein release assay, using the HER2/neu-overexpressing breast cancer cell line MDA-MB453, as target cells, and as effectors, PBMCs obtained from patients at diagnosis (n=34), and at the 12^th^ (n=31), and the 24^th^ (n=28) week of treatment. Calcein-acetomethoxy (AM) (Molecular Probes, Eugene, Oregon, USA)-labeled 10,000 target cells (target cells/patients PBMCs, ratio 30:1) were plated in triplicates into 96-well plates after treatment with trastuzumab at 20 μg/ml for 1 hour in ice (Roche, Basel, Switzerland). After 4 h at 37°C and 5% CO2, the release of Calcein (excitation = 485 nm; emission = 530 nm) was measured with a fluorescence plate reader (SpectraFluor Plus, Tecan, Männedorf, Switzerland). Maximal and spontaneous Calcein release values were obtained by adding either 100 µl lysis buffer (NaBO_3_ 0.025 M, Triton X-100 0.1%, pH 9) or HBSS, to wells containing 1 × 10,000 labeled target cells. The percentage of calcein release (CalR) was calculated as follow: CalR (%) = (experimental CalR– F_0_/F_max_ – F_0_) x100, where F_max_ referred to the maximal calcein released after the lysis of cells by adding 100 μl lysis buffer to the medium, and F_0_ referred to spontaneous calcein released from cells in the medium.

NK cell percentage from PBMCs count was obtained by flow cytometry protocols, across multiple time points: at diagnosis; 12^th^- 24^th^-week; 2-, 6- and 12-months follow-up. NK cells were labeled with α-CD3 phycoerythrin-texas red (mouse IgG1, clone UCHT1; Beckman Coulter), α-CD16 FITC (mouse IgG1, 3G8; Beckman Coulter), and α-CD56 PE (mouse IgG1 k, B159; BD Biosciences) antibodies and quantified on Cytomics FC500 flow cytometer (Beckman Coulter, Fullerton, CA, USA) with the CXP software (Beckman Coulter).

Finally, the percentage of cell lysis [lysis (%)], was assessed by using the CalR (%) normalized for 10,000 NK cells: Normalized ADCC=[CalR(%)*10,000]/[30*10,000*NKcell(%)], where 10,000 is the number of target cells in each well, 30 corresponds to the Effector: Target ratio and NK cell(%) is the percentage of NK cells quantified at the specific time point.

Genotyping at the FcγR locus was performed on genomic DNA by polymerase chain reaction (PCR) followed by direct sequencing to determine Single Nucleotide Polymorphisms (SNPs) variants at FcγR3A-158 and FcγR2A-131. The FcγR3A-158, indicated as V>F variant, was investigated through a nested PCR first using the forward primer 5′-TTGAAGGCCATGCTCAGTAAT-3′ and the reverse primer 5′-AGGCTGGTGCTACAGAACCTA-3′ to amplify a fragment of 1699 bp; and then the forward 5′-TTACAGAATGGCAAAGGCAG-3′ and the reverse 5′-TCTCCTCCCAACTCAACTTCC-3′ primers, to generate a 238 bp fragment. The FcγR2A-131, H>R variant, was analyzed through a single PCR using the forward primer 5′-CTGGTCAAGGTCACATTCTTC-3′ and the reverse 5′-CAATTTTGCTGCTATGGGC-3′ (277 bp fragment). The PCR products were purified and directly sequenced using the BigDye Terminator sequencing kit and an ABI Prism 3100 sequencer (both from Applied Biosystems, Foster City, CA).

### PCR-SSP KIR Typing

Genomic DNA was used to determine the genotype of the 13 functional KIR genes and the 2 pseudogenes by sequence-specific primer (SSP) polymerase chain reaction (PCR). Our laboratory had developed the procedure to consent multiplex PCR combinations of KIR pairs (17). Primers were assigned to 15 multiplex reactions, resulting in 30 amplicons that allowed the detection of all KIR genes as previously reported (17). The amplified products were analyzed on a 4% agarose gel and photo-documented.

### Frequencies of Alleles, Genotypes, and Haplotypes

KIR gene profiles were determined by the presence or absence of each KIR gene (KIR2DL1, KIR2DL2/KIR2DL3, KIR2DL4, KIR2DS2, KIR2DS3, KIR2DS4 (-full and -del variant), KIR2DS5, KIR2DL5, KIR2DS1, KIR3DL1/KIR3DS1, KIR3DL2, and KIR3DL3), in a given individual. All genotypes contained KIR2DL4, KIR3DL2, and KIR3DL3 as framework genes. Carrier frequencies of KIR genes and genotypes were calculated as their percentage of the total number of individuals. The frequencies of genes or haplotypes were calculated by direct counting, alleles duplicated on a single haplotype were not included, and absence was counted as a distinct allele. The composition and frequencies of haplotypes were determined using the Haplotype Analysis software (Forest Genetics and Forest Tree Breeding, Georg-Augst University Goettingen, Germany, distributed by the authors Eliades N-G., Eliades D. G)

In the assessment of the KIR genotype, group B genotypes were defined by the presence of one or more of the following genes: KIR2DL5, KIR2DS1, KIR2DS2, KIR2DS3, KIR2DS5, and KIR3DS1. Conversely, stable group A genotype was defined by the absence of all these genes and by the presence of KIR3DL1, KIR2DL1, KIR2DL3, and KIR2DS4 genes.

Moreover, centromeric (Cent) and telomeric (Tel) regions split in half the KIR genotype; KIR3DL3 and KIR3DP1 delimited the centromeric part of the KIR locus, whereas KIR2DL4 and KIR3DL2 delimited the telomeric part. KIR2DL5, KIR2DS5, and KIR2DS3 genes can be present both in centromeric and telomeric locations.

### HLA Typing

HLA genotyping was performed using PCR-sequence-based typing (PCR-SBT) of encompassing the 2-3 exons of HLA-A, HLA-B, HLA-C with primers specific for each class I locus as previously reported (17). PCR products were sequenced on an Applied Biosystems 3130 automated sequencer (Applied Biosystems, Foster City, CA, USA). The sequences were assembled in pairs and identified with the Sequence Pilot software (JSI medical systems, Germany). The frequencies of alleles were calculated by direct counting, and the number observed was divided by 2N (alleles duplicated on a single haplotype were not included, and absence was counted as a distinct allele).

### KIR/HLA Interactions

The associations of KIRs with their cognate HLA ligand were established based on predicted KIR/HLA combinations and according to the standard classification of HLA-C1, -C2, Bw4, and HLA A*03 and *11 based on the amino acid sequences determining the KIR-binding epitope (16, 23). Briefly, regarding HLA-C molecules, the KIR-ligand were classified as either HLA-C1 or HLA-C2 based on dimorphisms at amino acid positions 77 and 80 and Bw4 –I (Isoleucine) or Bw4-T (Threonine) based on substitutions at position 80. KIR 2DL1/2DS1, 2DS5, bound HLA-C2, KIR2DS4 the HLA allele-specific C*02:02, C*04:01, C*05:01, C*01:02, C*14:02, C*16:01, KIR2DL2, KIR2DL3, KIR2DS2, and KIR2DS3 the HLA-C1, KIR3DL1 and KIR3DS1 the HLA-Bw4 and KIR3DL2 the HLA allele-specific A*03 and A*11. The ligand for some KIRs (i.e.KIR2DL5 and KIR3L3) is still unknown. Non-classical HLA ligands, HLA-G, E, and F were not considered in the present analysis.

### Statistical Analysis

The genotypes of the KIR gene and KIR-HLA pair frequencies were individually determined by direct counting of the individual who tested positive for a specific (pair of) gene. Patients included in the study were all Caucasian. Differences in frequencies were estimated by using Yates-corrected Chi-square, Fisher’s exact test, and Jonckheere-Terpstra trend test for categorical variables. Haplotypes were determined by using the Haplotype Analysis software (Forest Genetics and Forest Tree Breeding, Georg-Augst University Goettingen, Germany). The Simpson Diversity Index was applied to evaluate the variability of haplotypes within pCR and partial pathological response (pPR) groups. T-test, Anova, and Kruskal-Wallis tests were used for non-parametric values. Kaplan-Meier method was used to analyze disease-free survival (DFS) and overall survival (OS) time from surgery (MedCal software package). A p-value of <0.05 was considered significant.

## Results

### Patients and Tumor Characteristics

Thirty-six women (median age 46 years, range 24-72, 23 (63.9%) under age 50) affected by a HER2-overexpressing locally advanced breast cancer were consecutively included in the present study. **Table 1** shows the major clinical parameters of the global case study. Almost one-half of patients had a hormone receptor-negative tumor (47.2%), and the majority were classified as stage IIB (63.9%). Twenty patients (20/36, 58.3%) had undergone radiotherapy; breast-only radiotherapy was administered in 16 patients, 4 patients with more than 3 positive-lymph nodes received additional radiotherapy for axilla. A patient undergoing axillary radiotherapy had completed the subsequent adjuvant chemotherapy protocol consisting of 3 cycles of Paclitaxel and trastuzumab and then trastuzumab alone for one year, the remaining 3 patients received another treatment with anthracycline-containing chemotherapy(ECx4). All tumors showed a ductal histotype (not shown). According to Response Evaluation Criteria in Solid Tumors (RECIST) criteria, a total of 16 pCR (44.4%) were achieved after neoadjuvant chemotherapy. The breast-conserving surgery rate was 38.9% (14/36). The induction of a pCR statistically was not correlated with younger than 50 years (P=0.096), hormone receptor status (P=0.576), or tumor stage (P=0.471). Due to the potential prognostic impact of pCR and to possibly identify a biomarker of pCR induction, for immunogenetic analyses, we divided patients into 2 groups: those achieving a pCR (n=16) compared to individuals reaching a pPR (n=20) after NC. The limited number of cases (n=2) with a borderline IHC score (IHC 2+) compared to those with a strong IHC score (IHC 3+, n= 34) does not allow us to make further categorial subgroup analyses to identify genetic characteristics of patients who have not achieved a pCR.

**Table 1:** Patients and tumor characteristics.

Clinical characteristics	Data
**Age**	
*< 50 years*	23 (63.9%)
*≥ 50 years*	13 (36.1%)
**Hormone Receptor status** *n (%)*	
ER+ and PgR+	12 (33.3)
ER+ and PgR-	6 (16.7)
ER- and PgR+	1 (2.8)
ER- and PgR-	17 (47.2)
**Tumor stage** *n (%)*	
IIA	3 (8.3)
IIB	23 (63.9)
IIIA	10 (27.8)
**Pathological response** *n (%)*	
pCR	16 (44.4)
pPR	20 (55.6)
**Surgery** *n (%)*	
Mastectomy	22 (61.1)
Conservative treatment	14 (38.9)

ER, estrogen receptor; PgR, progesterone receptor; pCR, pathological Complete Response; pPR, pathological Partial Response.

### KIR Haplotyping

**Figure 1:**
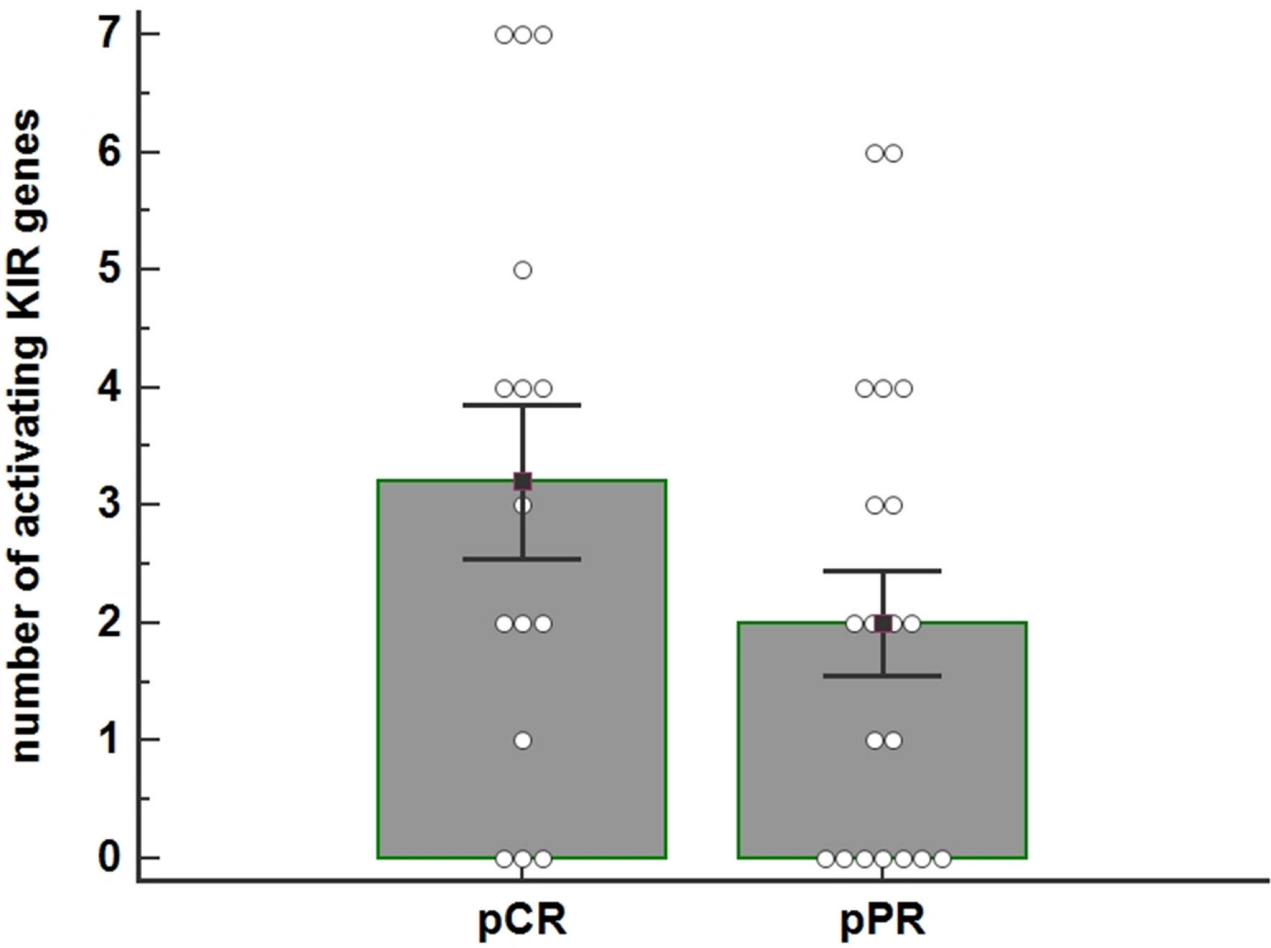
The histogram shows the average number of activating KIR genes for each individual as a function of the pathological Complete Response (pCR) (n=16) and pathological Partial Response (pPR) (n=20) to Neoadjuvant Chemotherapy (NC) treatment ( P=0.125). The number of activating KIRs appeared higher in patients who achieved pCR. P-value was calculated using the ANOVA test. Bar height and error bars represent the mean ± standard error mean (SEM) for the data set.

**Figure 2:**
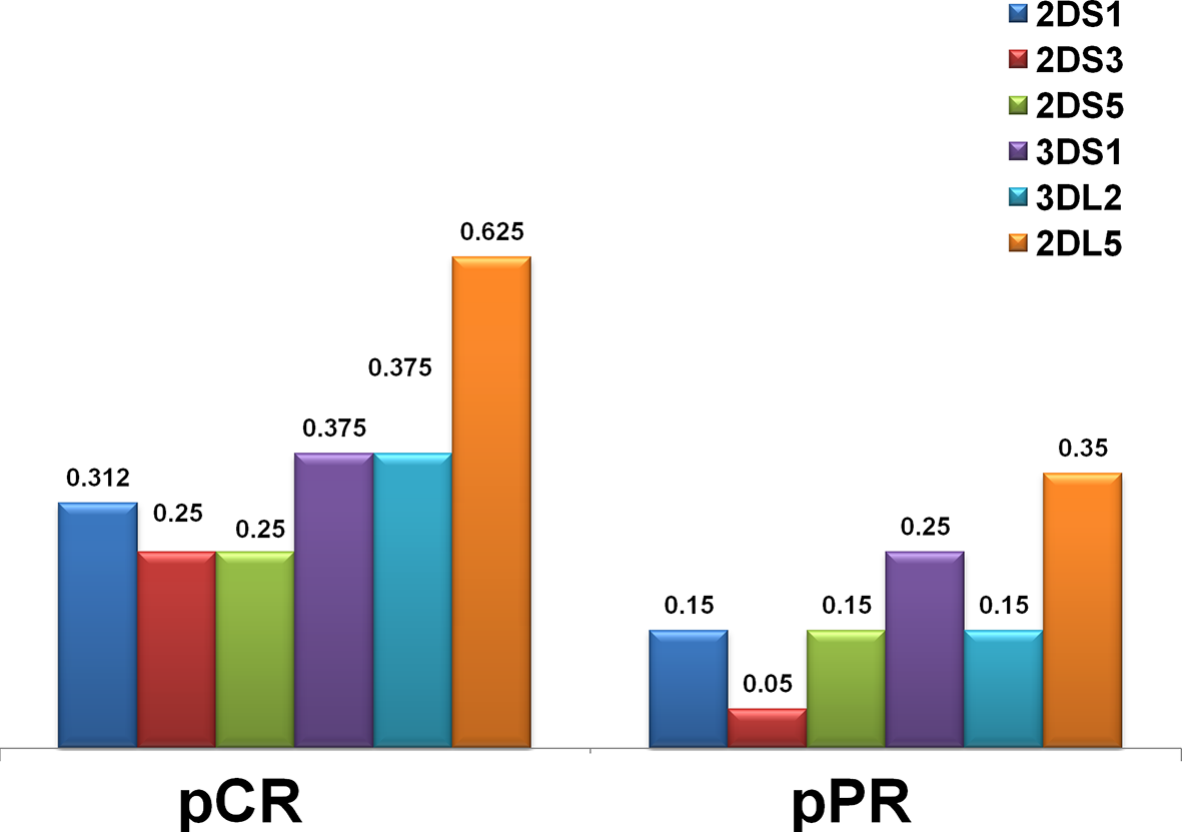
Distribution of KIR genes by frequency in the HER2-positive breast cancer in complete (pCR) and partial (pPR) response to neoadjuvant chemotherapy. The frequency of KIR activators (2DS1, 2DS3, 2DS5, 3DS1) was higher in patients who had a pCR. Framework gene KIR 3DL2, as well as a unique inhibitory KIR 2DL5 gene for which a definite ligand is still unknown, were also more frequent in the pCR group.

We determined the KIR gene for all 13 KIR genes, including the KIR2DL4full and KIR2DL4del variants, and 3 framework KIRs. The frequency of KIR genes was compared between 16 pCR and 20 pPR cases (**Table 2** and **Figure 1**). Framework genes (KIR3DL3, KIR3DL2 and KIR2DL4) were presented in all cases. KIR2DL1 and KIR2DL3 (centromeric region) were present in all pCR cases, KIR2DS3 and KIR2DL5 (both centromeric and telomeric) and KIR2DS1, KIR2DS3, KIR2DS5, and KIR3DS1 (centromeric) were more presents in the pCR cases, while KIR2DS4full variants (telomeric) showed a lower frequency in the pCR cases compared to pPR **(** **Figure 2**). However, none of the KIR genes reach a significant difference in the frequencies between patients achieving a pCR response compared to those with pPR.

**Table 2:** Comparison of the frequencies of KIR genes in NC treatment response.

KIR gene	CR (n=16)		RP (n=20)			Chi-square	P value*
**A haplotype associated**							
2DL1	16	(100.0%)	18	(90.0%)		–	
2DL3	16	(100.0%)	18	(90.0%)		–	
3DL1	15	(93.8%)	20	(100.0%)		–	
2DS4	15	(93.8%)	20	(100.0%)		–	
2DS4full	4	(25.0%)	8	(40.0%)		0.2387	0.625150
2DS4del	13	(81.3%)	17	(85.0%)		0.1606	0.688614
2DS4fulldel	15	(93.8%)	20	(100.0%)		–	
**B haplotype associated**							
2DS1	7	(43.8%)	6	(30.0%)		0.3728	0.541483
2DS2	9	(56.3%)	10	(50.0%)		0.0355	0.850554
2DS3	5	(31.3%)	2	(10.0%)		1.6406	0.20024
2DS5	6	(37.5%)	5	(25.0%)		0.2912	0.589465
3DS1	8	(50.0%)	7	(35.0%)		0.4693	0.493329
2DL2	9	(56.3%)	10	(50.0%)		0.0355	0.850554
2DL5	10	(62.5%)	7	(35.0%)		2.2900	0.130213
2DS4full	4	(25.0%)	8	(40.0%)		0.2387	0.625150
2DS4del	13	(81.3%)	17	(85.0%)		0.1606	0.688614
2DS4fulldel	15	(93.8%)	20	(100.0%)		–	

2DS4full: KIR2DS4 full-length variant, 2DS4del: KIR2DS4 deleted; variant, 2DS4fulldel: both KIR2DS4 full-length and deleted variant.

–not valuable for statistic analysis,*P Yates’correction,value < 0.05 was considered as statistically significant.

We observed 16 different KIR haplotypes in the population, of them 5 were present only in pCR (i.e. haplo-6, haplo-10, haplo-12, halo-15, haplo-16) and 4 were present only in pPR (i.e. haplo-1, haplo-2, haplo-4, haplo-13) (**Table 3**). The distribution of overall haplotypes was different between the two groups and pPR showed a reduced number of different haplotypes compared to pCR (Simpson Diversity Index: 0.06 for pPR and 0.03 for pCR).

**Table 3:** Comparison of KIR haplotype frequencies in NC treatment response.

cent	tel	Haplotype	pCR	pPR															
AB	AA	haplo-10	1	0	KIR2DS2	KIR2DL2	KIR2DL3	KIR2DL1	KIR3DL1	KIR2DS4full							KIR2DL4	KIR3DL2	KIR3DL3
AB	BB	haplo-15	1	0	KIR2DS2	KIR2DL2	KIR2DL3	KIR2DL1				KIR3DS1	KIR2DS1	KIR2DL5	KIR2DS3	KIR2DS5	KIR2DL4	KIR3DL2	KIR3DL3
AA	AB	haplo-6	1	0			KIR2DL3	KIR2DL1	KIR3DL1		KIR2DS4del	KIR3DS1		KIR2DL5		KIR2DS5	KIR2DL4	KIR3DL2	KIR3DL3
AB	AB	haplo-12	2	0	KIR2DS2	KIR2DL2	KIR2DL3	KIR2DL1	KIR3DL1		KIR2DS4del			KIR2DL5	KIR2DS3		KIR2DL4	KIR3DL2	KIR3DL3
AB	AB	haplo-16	2	0	KIR2DS2	KIR2DL2	KIR2DL3	KIR2DL1	KIR3DL1		KIR2DS4del	KIR3DS1	KIR2DS1	KIR2DL5	KIR2DS3		KIR2DL4	KIR3DL2	KIR3DL3
BB	AA	haplo-1	0	1	KIR2DS2	KIR2DL2			KIR3DL1	KIR2DS4full							KIR2DL4	KIR3DL2	KIR3DL3
AB	AB	haplo-13	0	1	KIR2DS2	KIR2DL2	KIR2DL3	KIR2DL1	KIR3DL1		KIR2DS4del	KIR3DS1		KIR2DL5	KIR2DS3		KIR2DL4	KIR3DL2	KIR3DL3
BB	AB	haplo-2	0	1	KIR2DS2	KIR2DL2			KIR3DL1		KIR2DS4del	KIR3DS1	KIR2DS1	KIR2DL5	KIR2DS3		KIR2DL4	KIR3DL2	KIR3DL3
AA	AA	haplo-4	0	1			KIR2DL3	KIR2DL1	KIR3DL1	KIR2DS4full							KIR2DL4	KIR3DL2	KIR3DL3
AA	AB	haplo-7	2	2			KIR2DL3	KIR2DL1	KIR3DL1		KIR2DS4del	KIR3DS1	KIR2DS1	KIR2DL5		KIR2DS5	KIR2DL4	KIR3DL2	KIR3DL3
AA	AA	haplo-3	2	3			KIR2DL3	KIR2DL1	KIR3DL1		KIR2DS4del						KIR2DL4	KIR3DL2	KIR3DL3
AA	AB	haplo-8	1	1			KIR2DL3	KIR2DL1	KIR3DL1	KIR2DS4full		KIR3DS1	KIR2DS1	KIR2DL5		KIR2DS5	KIR2DL4	KIR3DL2	KIR3DL3
AB	AA	haplo-11	1	2	KIR2DS2	KIR2DL2	KIR2DL3	KIR2DL1	KIR3DL1	KIR2DS4full	KIR2DS4del						KIR2DL4	KIR3DL2	KIR3DL3
AB	AB	haplo-14	1	2	KIR2DS2	KIR2DL2	KIR2DL3	KIR2DL1	KIR3DL1		KIR2DS4del	KIR3DS1	KIR2DS1	KIR2DL5		KIR2DS5	KIR2DL4	KIR3DL2	KIR3DL3
AA	AA	haplo-5	1	3			KIR2DL3	KIR2DL1	KIR3DL1	KIR2DS4full	KIR2DS4del						KIR2DL4	KIR3DL2	KIR3DL3
AB	AA	haplo-9	1	3	KIR2DS2	KIR2DL2	KIR2DL3	KIR2DL1	KIR3DL1		KIR2DS4del						KIR2DL4	KIR3DL2	KIR3DL3
					**centromeric genes**				**telomeric genes**								**framework genes**		

KIR haplotypes are composed of different motifs that included centromeric (yellow color), telomeric (green color) and framework (blue color) regions. Each motif has a different content and arrangement of genes; genes present in the respective motif are shown in the grey boxes, genes encoding activating KIR are indicated by the “S” letter; and those for inhibitory receptor by the “L” letter.

KIR haplotypes were grouped in centromeric (cen) and Telomeric (tel) following the recommendations from the 2011 KIR workshop (24). Briefly, cent A was characterized by the presence of 2DL3 and 2DL1, and cent B by at least one of the genes 2DS2, 2DL2. Tel A was characterized by 3DL1 and only one activating KIR gene, the 2DS4, while tel B had several activating genes (i.e. 3DS1, 2DS1, 2DS3, and/or 2DS5).

Cent A genotype in homozygous (AA) was found in 43.7% (7/16) of pCR and 50.0% (10/20) of pPR. Tel A in homozygous (AA) in 37.5% (6/16) pCR and 65.0% (13/20) in pPR, respectively.

These data indicated a trend towards a higher number of activator KIR genes (tel B) in patients achieving a pCR; the difference between the two groups is nearly statistically significant (Fisher exact test, p=0.1787) (**Figure 1**).

### Comparisons of KIR Genes and Their Cognate HLA Ligands Between pCR and pPR Groups

All HLA genes characterizing the KIR-HLA functional units were analyzed in 35 patients (one HLA typing in the pCR group did not amplify with the standard PCR used) (**Table 4**). Results showed the lowest correlation between pCR and pPR for HLA-A*11 and HLA-C*04 (Chi-square 1.4583 and 0.8929, respectively). The Odds ratio calculated was 3.800 (95%CI 0.68-21.13, P=0.127), and 2.02 (95%CI 0.65-6.25, P=0.22) for HLA-A*11 and HLA-A*C04, respectively.

**Table 4:** Comparisons of KIR genes and their cognate HLA ligands between CR and PR group.

	pCR n=30		pPR n=40		Chi-square	P*
***KIR Ligands***						
HLA Bw4						
Ile80	10	33.33%	11	27.50%	0.0694	0.792147
T80	6	20.00%	8	20.00%	0.0911	0.762725
Bw6	14	46.67%	21	52.50%	0.0583	0.809150
HLA-A*11	5	16.67%	2	5.00%	1.4583	0.227195
HLA-A*23	0	0.00%	4	10.00%	–	–
HLA-A*24	3	10.00%	2	5.00%	0.1122	0.737676
HLA-A*32	2	6.67%	4	10.00%	0.0004	0.950861
HLA-A*03	2	6.67%	2	5.00%	0.0497	0.823558
						
HLA-B*51	4	13.33%	7	17.50%	0.0202	0.886915
HLA-B*46:01	0	0.00%	0	0.00%	–	–
HLA-B*73:01	0	0.00%	0	0.00%	–	–
C1	10	33.33%	14	35.00%	0.0119	0.913175
C2	10	33.33%	11	27.50%	0.0694	0.792147
HLA-C*04	9	30.00%	7	17.50%	0.8929	0.344695
HLA-C*04: 01	3	10.00%	4	10.00%	0.0000	1.00000
HLA-C*05:01	1	3.33%	1	2.50%	0.2681	0.604625
HLA-C*01:02	1	3.33%	0	0.00%	–	–
HLA-C*14:02	0	0.00%	0	0.00%	–	–
HLA-C*16:01	1	3.33%	1	2.50%	0.2681	0.604625
	**pCR n=15**		**pPR n=20**		**Chi-square**	P*
***Activating KIR/HLA***						
2DS1+/HLA-C2+	5	33.3%	3	15.0%	0.7595	0.383470
2DS2+/HLA-C1+ or A*1101+	6	40.0%	7	35.0%	0.0025	0.959730
2DS3+/HLA-C1+	4	26.7%	1	5.0%	3.055	0.080491
2DS4full+/HLA-A*11+ or C*02:02, C*04:01, C*05:01,C*01:02, C*14:02, C*16:01	2	13.3%	2	10.0%	0.0529	0.818052
2DS5+/HLA-C2+	5	33.3%	2	10.0%	1.6406	0.200240
3DS1+/HLA-Bw4IoT+, or B*51	7	46.7%	5	25.0%	0.9537	0.328772
***Inhibitory KIR/HLA***						
2DL1+/HLA-C2+	11	73.3%	11	55.0%	0.5736	0.448814
2DL2+/HLA-C1+ (and B*46:01 B*73:01)	6	40.0%	7	35.0%	0.0025	0.959730
2DL3+/HLA-C1+	11	73.3%	12	60.0%	0.2140	0.643654
3DL1+/HLA-Bw4IoT+	13	86.7%	14	70.0%	0.5705	0.450058
3DL2+/HLA-A*03+ or A*11	7	46.7%	4	20.0%	1.7262	0.188891

+, present, *Chi-square with Yates correction, Bw6 (Ser 77, Asn 80, Leu 81, Arg 82, e Gly 83) Bw4 (Asn 77, Ile 80, Ala 81, Leu 82 e Arg 83) with Bw4I= Isoleucine or Bw4T=Threonine at residue 80 respectively. HLA-C1 epitopes (Serine at position 77, Asparagine at position 80) HLA-C2 epitopes (Asparagine at position 77, Lysine at position 80).

Comparison of the numbers of KIR-HLA ligands combinations showed an increased frequency of 2DS3+/HLA-C1+ (chi-square 3.055), 2DS5+/HLA-C2+ (chi-square 1.6406), 3DS1+/HLA-Bw4+ (chi-square 0.9537) and 2DS1+/HLA-C2+ (Chi-square 0.7595) in the pCR group compared to pPR patients (**Table 4**). Overall these combinations exerted an activating NK function and KIR genes were included in the tel B genotype. Only the 3DL2+/HLA-A03+ or -A11+ combination led to a slight increase in the frequency of inhibitor-based interactions in the pCR group (chi-square 1.7262) (**Table 4**). None of these combinations alone reached a statistical significance.

### Comparisons of KIR/HLA Functional Haplotypes Between pCR and pPR Groups

Based on the above-reported results we performed haplotype analysis of functional KIR/HLA gene combinations including the KIR2DL5 gene, whose HLA ligand is unknown, and the 2 framework genes (i.e. KIR2DL4 and KIR3DL3).

We found 27 different haplotypes (**Table 5**): 11 haplotypes and 13 haplotypes were uniquely found in the samples of pCR and pPR groups, respectively; 11 haplotypes were shared between pCR and pPR patients. Results indicated a reduction in the number of haplotypes shared between pCR and pPR groups using functional haplotyping (n=11) compared to KIR gene haplotyping (n=25) (**Tables 5**, **3**). We added the extension –Func to the centromeric and telomeric genotypes to underline the KIR+/HLA+ functional variants.

**Table 5:** Distribution of functional KIR/HLA haplotypes between CR and PR groups.

CentFunc	TelFunc	Haplotype	pCR	pPR														
AA-FUNC	AA-FUNC	haplo-12	1	0			2DL3	2DL1		2DS4full						2DL4	3DL2	3DL3
AA-FUNC	AA-FUNC	haplo-3	1	0				2DL1	3DL1				2DL5			2DL4	3DL2	3DL3
AB-FUNC	AA-FUNC	haplo-22	1	0	2DS2	2DL2	2DL3		3DL1	2DS4full						2DL4	3DL2	3DL3
AA-FUNC	AB-FUNC	haplo-5	1	0				2DL1	3DL1		3DS1	2DS1	2DL5		2DS5	2DL4		3DL3
AA-FUNC	AB-FUNC	Fhaplo-13	1	0			2DL3	2DL1	3DL1		3DS1	2DS1	2DL5		2DS5	2DL4		3DL3
AA-FUNC	AB-FUNC	haplo-6	1	0				2DL1	3DL1		3DS1	2DS1	2DL5		2DS5	2DL4	3DL2	3DL3
AA-FUNC	AB-FUNC	haplo-10	1	0			2DL3		3DL1		3DS1		2DL5			2DL4	3DL2	3DL3
AB-FUNC	AB-FUNC	haplo-21	1	0	2DS2	2DL2	2DL3		3DL1		3DS1		2DL5	2DS3		2DL4		3DL3
AB-FUNC	AB-FUNC	haplo-27	1	0	2DS2	2DL2	2DL3	2DL1	3DL1		3DS1	2DS1	2DL5	2DS3		2DL4		3DL3
AB-FUNC	AB-FUNC	haplo-23	1	0	2DS2	2DL2	2DL3	2DL1				2DS1	2DL5	2DS3	2DS5	2DL4		3DL3
AB-FUNC	AB-FUNC	haplo-25	1	0	2DS2	2DL2	2DL3	2DL1	3DL1				2DL5	2DS3		2DL4	3DL2	3DL3
AA-FUNC	AA-FUNC	haplo-7	0	1				2DL1	3DL1	2DS4full						2DL4		3DL3
AA-FUNC	AA-FUNC	haplo-11	0	1			2DL3	2DL1								2DL4		3DL3
AA-FUNC	AA-FUNC	haplo-15	0	1			2DL3	2DL1	3DL1	2DS4full						2DL4		3DL3
AB-FUNC	AA-FUNC	haplo-18	0	1	2DS2	2DL2	2DL3									2DL4		3DL3
AB-FUNC	AA-FUNC	haplo-24	0	1	2DS2	2DL2	2DL3	2DL1	3DL1							2DL4		3DL3
AB-FUNC	AA-FUNC	haplo-19	0	1	2DS2	2DL2	2DL3						2DL5			2DL4		3DL3
BB-FUNC	AA-FUNC	haplo-17	0	1	2DS2	2DL2			3DL1							2DL4		3DL3
AA-FUNC	AB-FUNC	haplo-4	0	1				2DL1	3DL1		3DS1		2DL5			2DL4		3DL3
AA-FUNC	AB-FUNC	haplo-9	0	1			2DL3		3DL1		3DS1		2DL5			2DL4		3DL3
AA-FUNC	AB-FUNC	haplo-14	0	1			2DL3	2DL1	3DL1		3DS1	2DS1	2DL5		2DS5	2DL4	3DL2	3DL3
AA-FUNC	AB-FUNC	haplo-1	0	1				2DL1			3DS1	2DS1	2DL5		2DS5	2DL4	3DL2	3DL3
AB-FUNC	AB-FUNC	haplo-26	0	1	2DS2	2DL2	2DL3	2DL1	3DL1		3DS1	2DS1	2DL5		2DS5	2DL4	3DL2	3DL3
BB-FUNC	AB-FUNC	haplo-16	0	1	2DS2	2DL2							2DL5	2DS3		2DL4		3DL3
AA-FUNC	AA-FUNC	haplo-2	2	3				2DL1	3DL1							2DL4		3DL3
AA-FUNC	AA-FUNC	haplo-8	1	3			2DL3		3DL1							2DL4		3DL3
AB-FUNC	AA-FUNC	haplo-20	1	1	2DS2	2DL2	2DL3		3DL1							2DL4		3DL3
					**centromeric genes**					**telomeric genes**						**framework genes**		

KIR haplotypes are composed of different motifs that included centromeric (yellow color), telomeric (green color) and framework (blue color) regions. Each motif has a different content and arrangement of genes; genes present in the respective motif are shown in the grey boxes, genes encoding activating KIR are indicated by the “S” letter; and those for inhibitory receptor by the “L” letter.

Cent A-Functional genotypes in homozygous (AA-Func) were found in 60.0% (9/15) of patients achieving a pCR and in 65.0% (13/20) of patients achieving a pPR, moreover, 2 patients with pPR showed a Cent B- Functional genotype in homozygous (BB-Func).

Tel A- Functional genotypes in homozygous (Tel AA- Func), without any functional activating KIR genes in the telomeric region, excepted the KIR2DS4full/HLA-A11, were found in 46.7% (7/15) of pCR, and 70.0% (14/20) of pPR cases.

By analyzing the haplotypes associated only with patients achieving pCR and pPR separately, we observed that 3DL2+/HLA-A03+ or A11+ were associated specifically with pCR carrying the Tel AA- Func genotype (Fisher exact test 0.008). Moreover, while KIR3DL2+/HLA-A03+ or A11+ was observed in 37.5% (3/8 tel B-Func) of unique pCR, KIR3DL2+/ligand+ was observed in 50.0% (3/6 tel B-Func) of unique pPR patients and was absent in patients with haplotype shared between pCR and pPR groups. Overall KIR3DL2+/ligand was observed in 40% (6/15) of patients with pCR and 15% (3/20) of those with a pPR (all carrying a tel B-Func genotype) (Fisher exact test p= 0.1157).

### Effects of tel A-F Genotype, KIR3DL2+/HLA-3 or 11 and FcR Combinations on ADCC Efficiency

To examine whether functional KIR/HLA genes included in the telomeric tel region and FcR polymorphisms influence ADCC efficacy *in vitro*, we determine the correlation between specific KIR/FcR combinations and normalized ADCC levels obtained *in vitro* using PBMCs of the corresponding patients (9).

**Figure 3:**
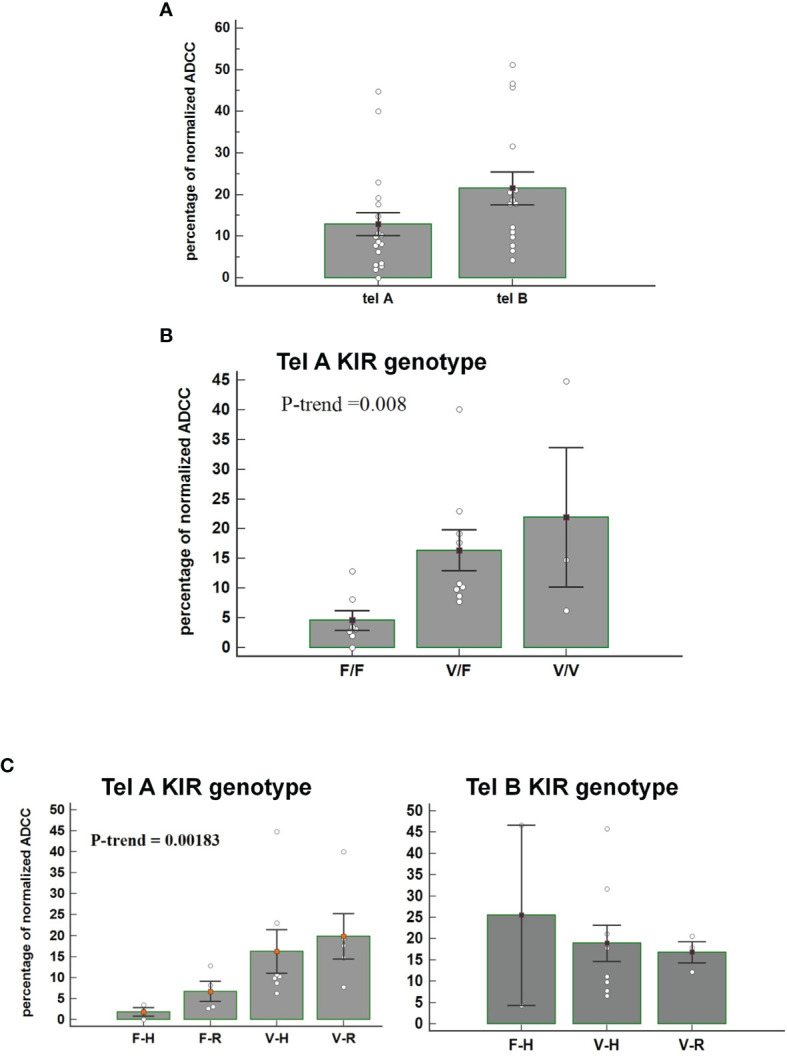
**(A)** Figure shows the percentage of normalized Antibody-Dependent Cell Cytotoxicity (ADCC) mediated by NK cells, carrying the tel A genotype and the tel B genotype, in the presence of trastuzumab. Each histogram represents the extent of normalized lysis of calcein-labeled MDA-MB453 cells in the presence of trastuzumab and 10,000 NK cells as effector cells. A higher ADCC efficacy is mediated by effector cells of patients carrying the tel B KIR genotype compared to those obtained from patients with tel A KIR genotype (P=0.07: Bar height and error bars represent the mean ± standard error of the mean (SEM) for the data set. The P-value was calculated using the ANOVA test. **(B)** Histograms represent the percentage of normalized ADCC mediated by NK cells of patients carrying the KIR tel A genotype (n=19) according to V and F polymorphic FcγRIII variants. Higher cytolysis was observed in patients carrying the tel A genotype in the presence of FcγRIII V-allele compared to F-allele. In detail, we observed the following percentage of normalized ADCC: 4.6 (95%CI -4.33-13.55) in the presence of the homozygous FcγRIII F variant (F/F); 17.06 (95%CI 8.70-25.43) in cases of heterozygous FcγRIII variants (V/F); and 21.9 (95%CI 8.3-35.6) for the homozygous FcγRIII V variant (V/V) carriers. The P value of Jonckheere-Terpstra trend was significant(p=0.0080). Bar height and error bars represent the mean ± standard error of the mean (SEM) for the data set. **(C)** Histogram report the percentage of normalized ADCC according to KIR telA versus telB genotype, and according to FcγR3A F>V and FcγR2A R>H polymorphic variants. A gradual increase of the percentage of normalized ADCC was observed in patients carrying the FcγR3A V polymorphism, with a higher affinity for the IgG1-Fc, and the inhibitory tel A genotype. The effect of the FcγR2A R variant, with a reduced affinity for IgG1-Fc, was limited. (Jonckheere-Terpstra trend test, p=0.0018). The percentages of normalized ADCC were respectively: 1.98 for FcγR3A- FcγR2A (F-H);, 5.62 for (F-R), 10.26 for (V-H); and 17.65 for (V-R). Conversly, normalized ADCC measured in patients carrying the tel B genotype showed that neither FcγR3A nor FcγR2A polymorphisms had a significant effect (Jonckheere-Terpstra trend test, p=0.8455). V= FcγR3A V/V or V/F genotypes; F= FcγR3A F/F genotype; H= FcγR2A H/H or H/V-genotypes; R= FcγR2A R/R-genotype. Each data represent three replicates. Bar height and error bars represent the mean ± standard error of the mean (SEM) for the data set.

We observed that patients carrying the telomeric tel B- Func genotype, which includes several activating KIR, had a better ADCC efficacy than patients with the tel A- Func genotype [21.53 (95%CI 13.09-30.01) vs 12.92 (7.08-18.74), t-test p=0.07] (**Figure 3A**) and almost all combined with the FcγRIII V-allele (Tel B-F 12/14, 85.7%; Tel-A 7/19, 36.8%, Fisher exact test P= 0.0113). The consequence was altogether a more evident effect of FcγRIII V-carrier on *in vitro* NK cytolysis; higher cytolysis was observed in patients carrying the tel A-Func genotype in the presence of FcγRIII V-allele (F/F 4.6 [95%CI -4.33-13.55]; V/F 17.06 [95%CI 8.70-25.43]; V/V 21.9 [8.3-35.6] Jonckheere-Terpstra trend test, p=0.008) (**Figure 3B**).

The effect of FcR polymorphisms on ADCC was also evident by combining the FcγR3A and the FcγR2A polymorphic variants. Fcγ receptors differ in their affinity for the antibody Fc-fragment and cell-type expression; FcγR2A is mainly expressed on NK cells and monocytes, while the FcγR2A is expressed on monocytes, platelets, neutrophils, macrophages, and dendritic cells, but not on lymphocytes. FcγR3A had two variants, the FcγR3A-V and FcγR3A-F, and NK cells expressing the V variant demonstrated a stronger binding affinity for Ig-FC than the F-variant. FcγR2A has two, variants (R131 and H131), which differ at amino acid position 131 in the extracellular domain with the FcγR2A-H variant showing a higher binding affinity for IgG1 and IgG2 than the R-variant (25). We observed a gradual increase in the ADCC efficacy, from FcγR3A-F/FcγR2A-H to FcγR3A-V/FcγR2A-R combination of FcγRIII and FcγR2A variants, that resulted statistically significant only in patients lacking the activating KIRs (tel A-F) (**Figure 3C**).

**Figure 4:**
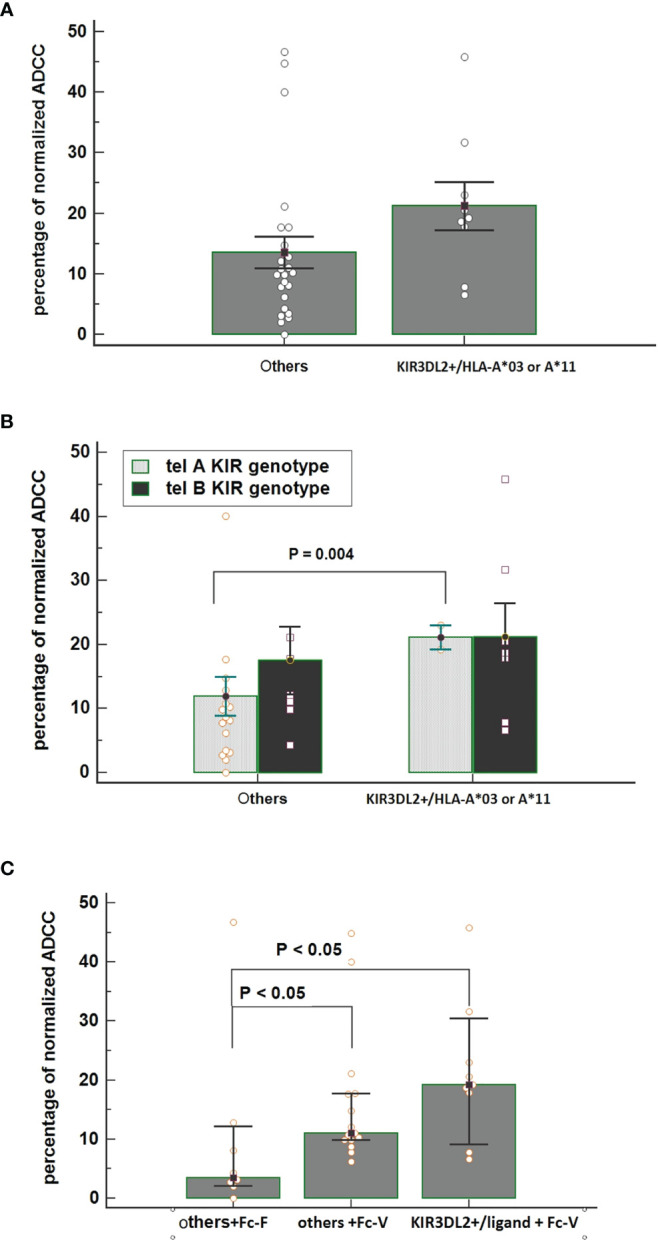
**(A)** Comparison of normalized Antibody-Dependent Cell Cytotoxicity (ADCC) mediated by NK cells obtained from patients carrying the functional KIR3DL2+/HLA-A*03 or A*11 combinations and from all of the other patients carrying other KIR/HLA combinations. Each histogram represents the extent of normalized lysis of calcein-labeled MDA-MB453 cells in the presence of trastuzumab and 10,000 NK cells as effector cells. Bar height and error bars represent the mean ± standard error of the mean (SEM) for the data set. A higher ADCC efficacy is mediated by effector cells of patients carrying the KIR3DL2+/ligand compared to effectors obtained from patients characterized by other KIR/HLA combinations (ANOVA test, P = 0.036). **(B)** Interactive action of KIR tel A or B genotype and KIR/HLA combination on ADCC efficacy showed by two ways Anova. A significant increase in cytolysis was observed by using effector cells obtained from patients carrying the tel A-Func genotype in the presence of KIR3DL2+/HLA-A*03 or A*11 compared to those obtained from patients with other KIR/ligand combinations. [% ADCC 11.94 (95%CI 5.58-18.31); % ADCC 21.10 (95%CI 2.54-39.65), t-test p=0.004]. Bar height and error bars represent the mean ± standard error of the mean (SEM) for the data set. **(C)** An increased effect on ADCC efficacy was observed in the presence of effector cells obtained from patients carrying both the FcγR3A V-allele (Fc-V) and the KIR3DL2+/HLA-A03 or A*11 compared to those carrying the FcγR3A F-allele (Fc-F) and other KIR/HLA combinations [% ADCC =3.47 vs 19.21, p<0.05]. A significant difference was also observed between other KIR/Ligand combinations according to the FcγR3A allele [% ADCC others+Fc-F = 3.47 others +Fc-V= 11.06, Kruskal-Wallis test P <0.05].

Interestingly, all patients carrying matched KIR3DL2+/ligand+ showed the FcγR3A V-carrier (n=9); PBMCs collected from these patients before treatment showed a higher ADCC efficacy compared with PBMCs collected from patients lacking this combination (**Figure 4A**). Thus, the highest increase in ADCC efficacy was noticed for PBMCs obtained from patients having the combination of both Tel A-Func and KIR3DL2+/ligand compared to those carrying the Tel A-Func with other KIR/HLA combinations (**Figure 4B**). The increase in cytolysis was also associated with the presence of the FcγR3A-V allele (**Figure 4C**). These results underlined the key role played by NK cell receptors as FcγR3A-V variant and specific KIR receptors in determining a better trastuzumab ADCC efficacy, while suggesting a minor role for receptors presented by other ADCC mediators, as the FcγR2A, predominantly expressed on monocytes and neutrophils.

### Analysis of CD16 and CD56 Expression Levels on NK Cells During Adjuvant Treatment According to patients KIR tel and KIR3DL2+/HLA Genotypes

We then investigated the potential contribution of KIR tel and KIR3DL2+/HLA genotypes on biological and clinical parameters measured after surgery during adjuvant chemotherapy and at follow-up.

**Figure 5:**
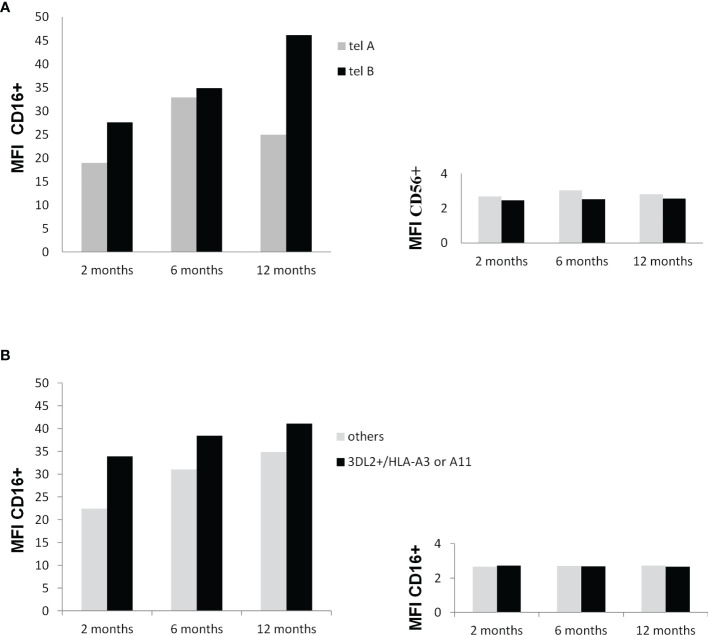
**(A)** Influence of the KIR tel B genotype on CD16 and CD56 membrane expression at 2, 6, and 12 months after surgery. Mononuclear peripheral blood cells from KIR tel genotyped patients (n=10) were labeled with anti-CD3 and anti-CD16 monoclonal antibody and anti-CD56 antibody and analysed by flow cytometry. An increase in the expression of CD16 on NK cells carrying the tel B genotype was found during time (trend P=0.11) **(A)** Influence of the potential functional KIR3DL2+/HLA-A*03 or A*11 gene combinations on CD16 and CD56 membrane expression at 2, 6, and 12 months after surgery. Mononuclear peripheral blood cells from KIR tel genotyped patients were labeled toghther with anti-CD3, anti-CD16 , and anti-CD56 antibodies and analysed by flow cytometry. Differences among groups were not statistically significant.

As shown in **Figures 5A, B**, the expression levels of CD16, a marker of NK cell mature status, increased overtime in particular in patients carrying the tel B genotype, while the change in the CD56 expression levels on NK cells was inconsistent. The presence of KIR3DL2+/HLA-A03 or A11 gene combinations on the CD16 and CD56 expression levels had no substantial effect during the time.

### Influence of Haplotype tel and KIR3DL2+/HLA-3 or 11 on Clinical Outcome

**Figure 6:**
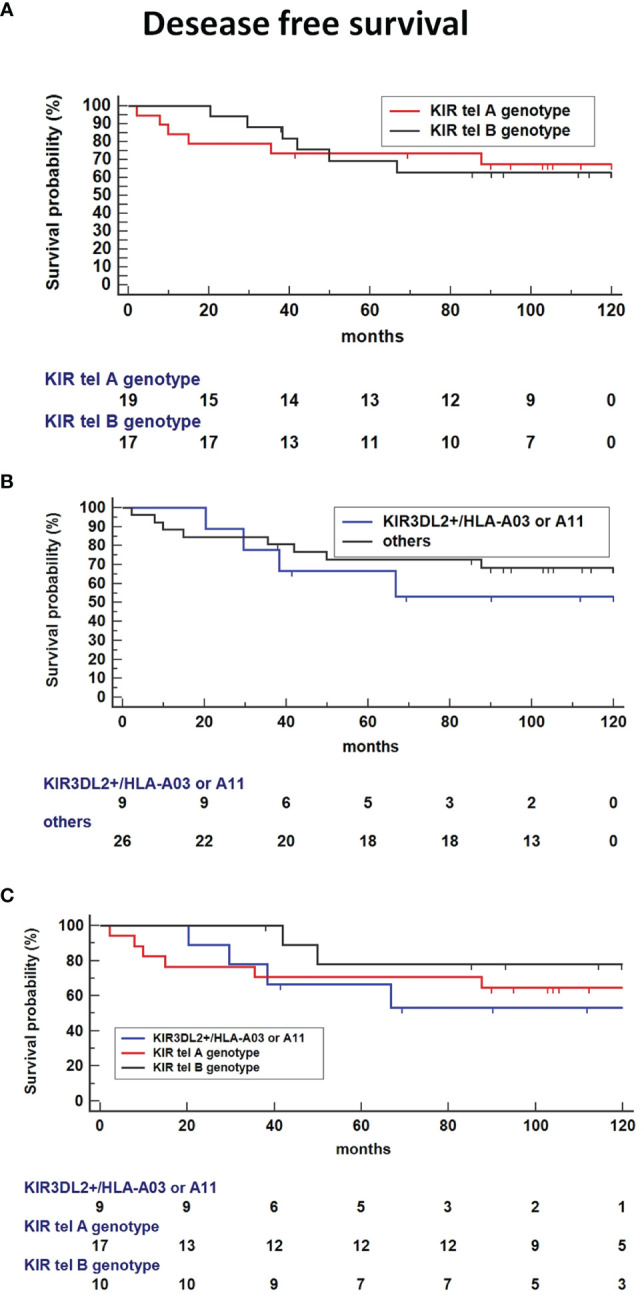
Kaplan-Meier survival analysis showed improved Disease-free survival (DFS) time for tel B genotype carriers compared to tel A carriers **(A)** and absence of the functional tel KIR3DL2+/HLA-A*03 or A*11 combinations compared to other KIR/HLA combinations **(B)**. **(C)** Co-presence of Tel B genotype and KIR3DL2+/HLA-A*03 or A*11 genes reduce DFS compared to Tel-B lacking the KIR3DL2+/HLA-A*03 or A*11 combinations (HR: 3.85, 95%CI 0.72-20.5, p = 0.1141).

Kaplan-Meier survival analysis showed no difference in DFS time for tel B genotype carriers compared to tel A carriers (**Figure 6A**). Interestingly, the absence of the functional tel KIR3DL2+/HLA-A3 or 11 combinations seemed to decrease the DFS compared to other KIR/HLA combinations (**Figure 6B**). The presence of both Tel B genotype and KIR3DL2+/HLA-A3 or 11 genes reduces DFS compared to Tel-B lacking the KIR3DL2+/HLA-A3 or 11 combinations (HR: 3.85, 95%CI 0.72-20.5, p = 0.1141(**Figure 6C**).

**Figure 7:**
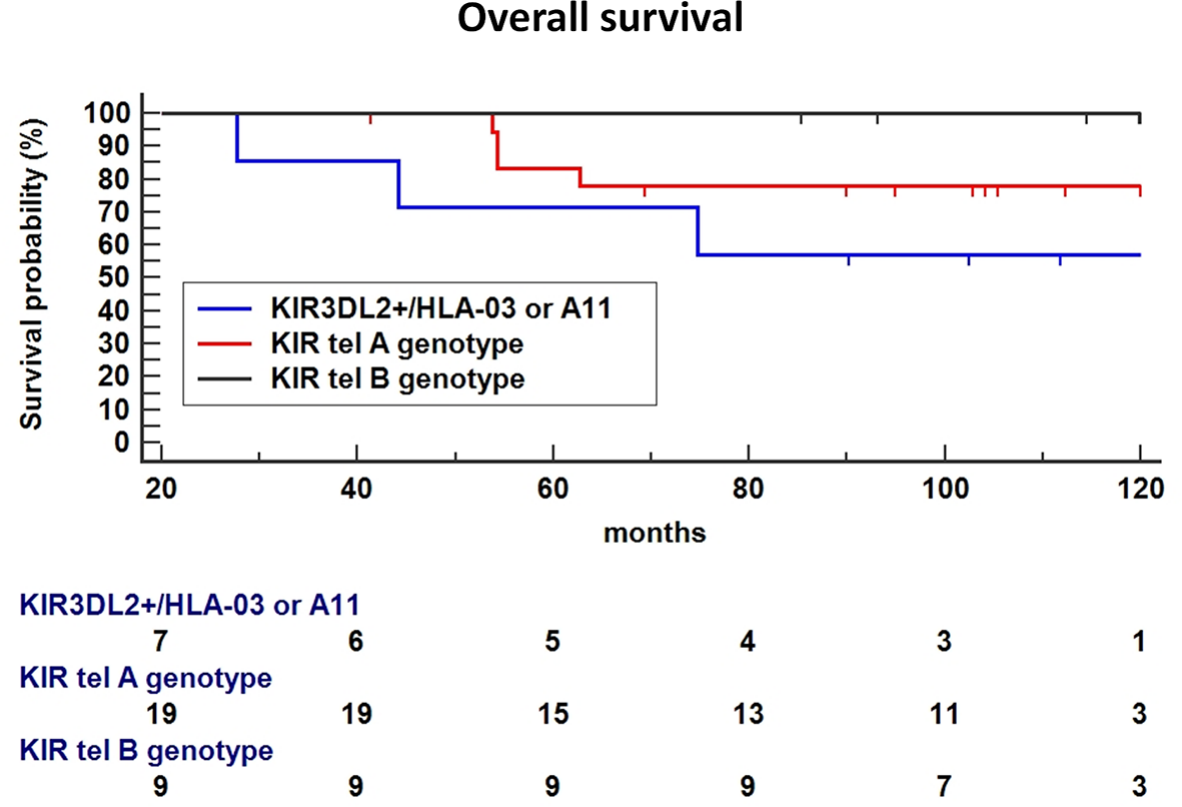
In Breast Cancer (BC) patients treated with Neoadjuvant Chemotherapy (NC), the haplotype including the functional tel B genotype and the KIR3DL2+/ligand was significantly associated with a poor Overall Survival (OS) compared to tel B haplotype without the 3DL2+/ligand as the reference (P = 0.0328), a trend toward a lower-survival was found using Tel A without the 3DL2+/ligand combination as the reference (HR: 2.66, 95%CI 0.47-14.8, P=0.26).

Finally, the haplotype including the functional tel B genotype and the KIR3DL2+/ligand was significantly associated with a poorer OS compared to tel B haplotype without the 3DL2+/ligand as the reference (P = 0.0328, **Figure 7**), or Tel A without the 3DL2+/ligand combination as the reference (HR: 2.66, 95%CI 0.47-14.8, P=0.26).

## Discussion

Response to trastuzumab treatment in cancer is mainly mediated by ADCC (1), as a consequence, a reduced DFS has been hypothesized to be related to a less NK cell cytotoxic efficacy (8). However, the impact of KIRs involved in ADCC regulation in trastuzumab-treated BC has been poorly explored (26).

Key findings disclosed herein supported a role of the immune genetic background of KIRs in the efficacy of trastuzumab treatment in HER2-positive BC. Indeed, although preliminary, our data showed that the KIR functional repertoire could be associated both with the response to neoadjuvant therapy and with DFS and OS in the adjuvant setting. KIR haplotypes, HLA ligands for KIRs, and combinations of KIR and HLA were investigated in 35 patients with HER2-positive locally advanced BC patients treated with trastuzumab both given as NC together with paclitaxel, and for 1 year after surgery.

In this cohort of patients, no correlation was highlighted between the induction of a pCR and clinical parameters as hormone receptor status, age, and tumor stage. ER and PgR are expressed by a low number of cases, too exiguous to evaluate a possible correlation with a clinical response as reported elsewhere (3). Young age (<50 years) is considered as a favorable predictive factor for pCR in BC (27). Consistently, eleven patients aged under 50 years showed a higher rate of pCR compared with 5 patients in the older group, but the different frequencies did not reach a statistical significance. Finally, the tumor stage could not be considered an influencing variable in our study since all patients were characterized by a locally advanced stage (Stage II-III).

Through the KIR gene repertoire analysis towards a pCR after NC, we observed a higher frequency of activating KIRs present in the telomeric B KIR genotype, compared to patients who obtained a pPR. Furthermore, we found that the specific presence of the inhibitory functional KIR3DL2/HLA-A03 or A11 combination was more frequent in pCR patients. KIR haplotypes can be divided into two regions (i.e. centromeric, cen, and telomeric, tel) and in two main groups based on the different numbers and types of KIR genes. The “A” type includes mainly inhibitory KIRs while the type “B”, is characterized by the presence of at least one activating KIR receptor among KIR2DS1, KIR2DS3, KIR2DS5, and KIR3DS1 (16). Interestingly, we observed that individuals with a tel B KIR stimulatory haplotype at the time of diagnosis (including those with a KIR3DL2+/ligand combination) showed slightly higher ADCC activity *in vitro* than those with a tel A KIR inhibitory haplotype. We hypothesized that the prevalence of activating KIRs expressed by the tel B haplotype could favor the NK cell cytotoxicity thereby contributing to trastuzumab-mediated ADCC efficiency. Similar results have been reported for other tumors treated with targeted-antibodies acting through the ADCC mechanism. For example, the presence of the activating KIR2DS1 strongly potentiated rituximab-mediated ADCC in non-Hodgkin lymphoma (28); the presence of a stimulatory tel B genotype increased the anti-GD2-mediated ADCC in neuroblastoma (29). However, in other cases, the presence of inhibitory KIRs was shown to be necessary to obtain a clinical benefit with the tumor-specific antibody (30, 31).

There may be a synergic effect between the KIR haplotype and high-affinity FcγR variants, which are responsible for a higher affinity of CD16-positive NK cells to the Fc fragment of the monoclonal antibody (29). Patients with these specific Fc genetic variants had shown stronger ADCC efficacy and superior event-free survival (29). Similarly, we found an impact of the FcγR3A polymorphism in patients characterized by the tel A KIR haplotype. In particular, the presence of at least one FcγR3A V-allele (V/V or V/F genotypes), responsible for a stronger affinity of NK cells to trastuzumab, induced a higher ADCC efficiency compared to cases showing a FcγR3A F/F genotype within the tel A population, while no difference was observed for patients carrying a tel B haplotype. This observation was independent of the FcγR2A genotype since FcγR3A V-carriers showed a similar high ADCC, in the presence of both the H allele and the R allele of the FcγR2A. These data suggested that, among individuals carrying the stronger inhibitory KIR tel A genotype (showing lower NK ADCC efficiency *in vitro*), those having a FcγR3A V variant (with higher affinity for the Fc fragment of IgG) could partially compensate for their decreased function, possibly by increasing the binding to trastuzumab. Indeed, as we previously demonstrated, the presence of just one V allele in the FcγR3A locus significantly improved the trastuzumab-mediated ADCC activity (9).

Regardless of the tel A and tel B haplotypes, we noted a prevalence of the functional KIR3DL2+/HLA ligand combination, present in both haplotypes, in patients achieving a pCR. KIR3DL2 is also known as CD158k, and is the only one in the KIR family to be expressed as a disulfide-linked homodimer (32). The known HLA-specific ligands for this receptor are HLA-A3, -A11, and the free heavy chain form of HLA-B27 (16). Furthermore, KIR3DL2 binds to CpG oligonucleotides particularly abundant in microbial genomes, transporting them to the Toll-Like Receptor 9 (TLR9) to mount an innate immune response, including NK cell activation (33). Interestingly, the KIR3DL2 carrier patients in our cohort also had the FcγR3A V-allele, and, accordingly, exhibited greater ADCC efficiency than the KIR3DL2+ and FcγR2A F/F individuals. These data agreed with the findings described by Sun et al, that reported a higher NK cell-mediated cytolysis of Multiple Myeloma cells dependent on the mAb isatuximab in the presence of the KIR3DL2+, HLA-A3/A11+, and FcγR3A V markers (34).

Therefore, in the neoadjuvant setting, the characterization of the functional KIR repertoire together with the analysis of the FcγR3A polymorphism could favor the early identification of those patients who will respond better to trastuzumab, possibly contributing to the design of a more personalized therapy.

We investigated the KIR repertoire with biological and clinical parameters measured at follow-up. We noticed that individuals carrying the stimulatory tel B haplotype showed an increase in CD16 expression on NK cells 1 year after surgery, while there was no difference in patients with an inhibitory tel A haplotype. Likewise, Isitman and colleagues reported a higher frequency of CD16+ cells in the absence of inhibitory KIRs, thus suggesting that NK cells that mainly express inhibitory KIRs are poorer ADCC effectors than NK cells lacking these receptors (35). The increase of the CD16+ cell rate over time after treatment indicated the enrichment of an important fraction of mature NK cells, the only population of these cells expressing KIR receptors and functionally equipped to trigger ADCC in HER2-expressing tumor cells in the presence of trastuzumab. This is in agreement with other studies showing that the number of intratumoral and circulating CD16+ NK cells increased during mAb-based treatment as CD16-ligation positively affects NK cell survival and proliferation, and may be associated with a favorable patients’ outcome (36, 37). The role of these CD16+ cells is further supported by the observation that clinical response correlated with the presence of specific activating Tel B KIR genotype, while the CD56 expression on NK cells was found unchanged after treatment.

However, when comparing DFS we found no significant difference between BC patients with different KIR haplotypes. Conversely, the presence of the inhibitory KIR3DL2 appeared to be associated with worse DFS and OS, while patients with a tel B haplotype in the absence of KIR3DL2 showed the best prognosis. In particular, the expression of some other inhibitory KIR receptors correlated with a poor prognosis also in other pathologies, as previously observed also by our group (19, 23, 38, 39). Interestingly, KIR3DL2 expression on T-cells is a marker for several cutaneous T-cell lymphomas as Sésary syndrome and mycosis fungoides (40). Indeed, this KIR receptor is expressed not only on NK cells, but also by a small percentage of CD4+ and CD8+ T lymphocytes, and in lymphomatous T cells, and it represents a promising therapeutic target (40). In this context, KIR3DL2-targeted monoclonal antibody, IPH4102, has recently been developed for the treatment of these diseases demonstrating a safe profile and encouraging clinical activity in a phase I clinical trial (41). In our cohort of BC patients, we noted a worse outcome in the presence of KIR3DL2, thus suggesting that this molecule may be eligible as a target for further treatments in this setting as well as in others, although our data are preliminary for this purpose.

Our study has several limitations, as the small cohort of patients that did not allow us to perform further subgroup analyses evaluating the contribution of different KIR/ligand combinations, and the lack of a control group of healthy women to compare the KIR repertoires and the ADCC efficiency. Moreover, it should be noted that the treatment of patients also included paclitaxel, which is known to act on NK cells, modifying their cytolytic potential (42).

In conclusion, our data are preliminary but have suggested a potential predictive role for a specific KIR genotype, namely tel B, in identifying patients who will achieve pCR after NC, and have supported a negative prognostic impact of KIR3DL2/HLA-A03 or A11 in the adjuvant setting. Prospective future studies, involving larger series and multiple centers, need to be performed to confirm the present findings. Moreover, clinical interventions in patients with unfavorable KIR profiles could be envisaged by using NK immunomodulatory drugs or mAb, like the IPH4102 targeting the KIR3DL2+ cells, with standard chemotherapy.

## Data Availability Statement

The original contributions presented in the study are included in the article/Supplementary Material. Further inquiries can be directed to the corresponding author.

## Ethics Statement

The studies involving human participants were reviewed and approved by phase II CRO Clinical Trial, NCT02307227, Centro di Riferimento Oncologico, Aviano, Italy. The patients/participants provided their written informed consent to participate in this study.

## Author Contributions

EM and VD conceived the study and drafted the manuscript. EM and MDZ performed the experiments. GM, DL, SSc, and SSp collected and analyzed clinical data. SM performed surgical operations and contributed to analyzing data. TP performed histopathological diagnosis and immunohistochemistry. RD and AS supervised the study and reviewed the manuscript. VDR performed the statistical analysis and supervised the study. All authors read and approved the final manuscript.

## Funding

Centro di Riferimento Oncologico, Aviano, Italy

## Conflict of Interest

The authors declare that the research was conducted in the absence of any commercial or financial relationships that could be construed as a potential conflict of interest.

The editor PL declared a past co-authorship with one of the authors VR at the time of review.

## Publisher’s Note

All claims expressed in this article are solely those of the authors and do not necessarily represent those of their affiliated organizations, or those of the publisher, the editors and the reviewers. Any product that may be evaluated in this article, or claim that may be made by its manufacturer, is not guaranteed or endorsed by the publisher.
